# Susceptibility of influenza viruses to hypothiocyanite and hypoiodite produced by lactoperoxidase in a cell-free system

**DOI:** 10.1371/journal.pone.0199167

**Published:** 2018-07-25

**Authors:** Urmi Patel, Aaron Gingerich, Lauren Widman, Demba Sarr, Ralph A. Tripp, Balázs Rada

**Affiliations:** University of Georgia, College of Veterinary Medicine, Department of Infectious Diseases, Athens, Georgia, United States of America; Deutsches Primatenzentrum GmbH - Leibniz-Institut fur Primatenforschung, GERMANY

## Abstract

Lactoperoxidase (LPO) is an enzyme found in several exocrine secretions including the airway surface liquid producing antimicrobial substances from mainly halide and pseudohalide substrates. Although the innate immune function of LPO has been documented against several microbes, a detailed characterization of its mechanism of action against influenza viruses is still missing. Our aim was to study the antiviral effect and substrate specificity of LPO to inactivate influenza viruses using a cell-free experimental system. Inactivation of different influenza virus strains was measured *in vitro* system containing LPO, its substrates, thiocyanate (SCN^-^) or iodide (I^-^), and the hydrogen peroxide (H_2_O_2_)-producing system, glucose and glucose oxidase (GO). Physiologically relevant concentrations of the components of the LPO/H_2_O_2_/(SCN^-^/I^-^) antimicrobial system were exposed to twelve different strains of influenza A and B viruses *in vitro* and viral inactivation was assessed by determining plaque-forming units of non-inactivated viruses using Madin-Darby canine kidney cells (MDCK) cells. Our data show that LPO is capable of inactivating all influenza virus strains tested: H1N1, H1N2 and H3N2 influenza A viruses (IAV) and influenza B viruses (IBV) of both, Yamagata and Victoria lineages. The extent of viral inactivation, however, varied among the strains and was in part dependent on the LPO substrate. Inactivation of H1N1 and H1N2 viruses by LPO showed no substrate preference, whereas H3N2 influenza strains were inactivated significantly more efficiently when iodide, not thiocyanate, was the LPO substrate. Although LPO-mediated inactivation of the influenza B strains tested was strain-dependent, it showed slight preference towards thiocyanate as the substrate. The results presented here show that the LPO/H_2_O_2_/(SCN^-^/I^-^) cell-free, *in vitro* experimental system is a functional tool to study the specificity, efficiency and the molecular mechanism of action of influenza inactivation by LPO. These studies tested the hypothesis that influenza strains are all susceptible to the LPO-based antiviral system but exhibit differences in their substrate specificities. We propose that a LPO-based antiviral system is an important contributor to anti-influenza virus defense of the airways.

## Introduction

Influenza virus epidemics and periodic pandemics affect millions of people worldwide causing serious substantial morbidity and mortality representing a major economic burden [1, 2]. Current prophylactic options are problematic due to the yearly need for strain-specific vaccination, the development of drug resistance, and changes in the virus related to antigenic drift and viral reassortment [3–5]. Identifying novel antiviral approaches that are broadly effective against several influenza strains is urgently needed. In this regard, the respiratory innate immune system could possess such mechanisms.

**Fig 1:**
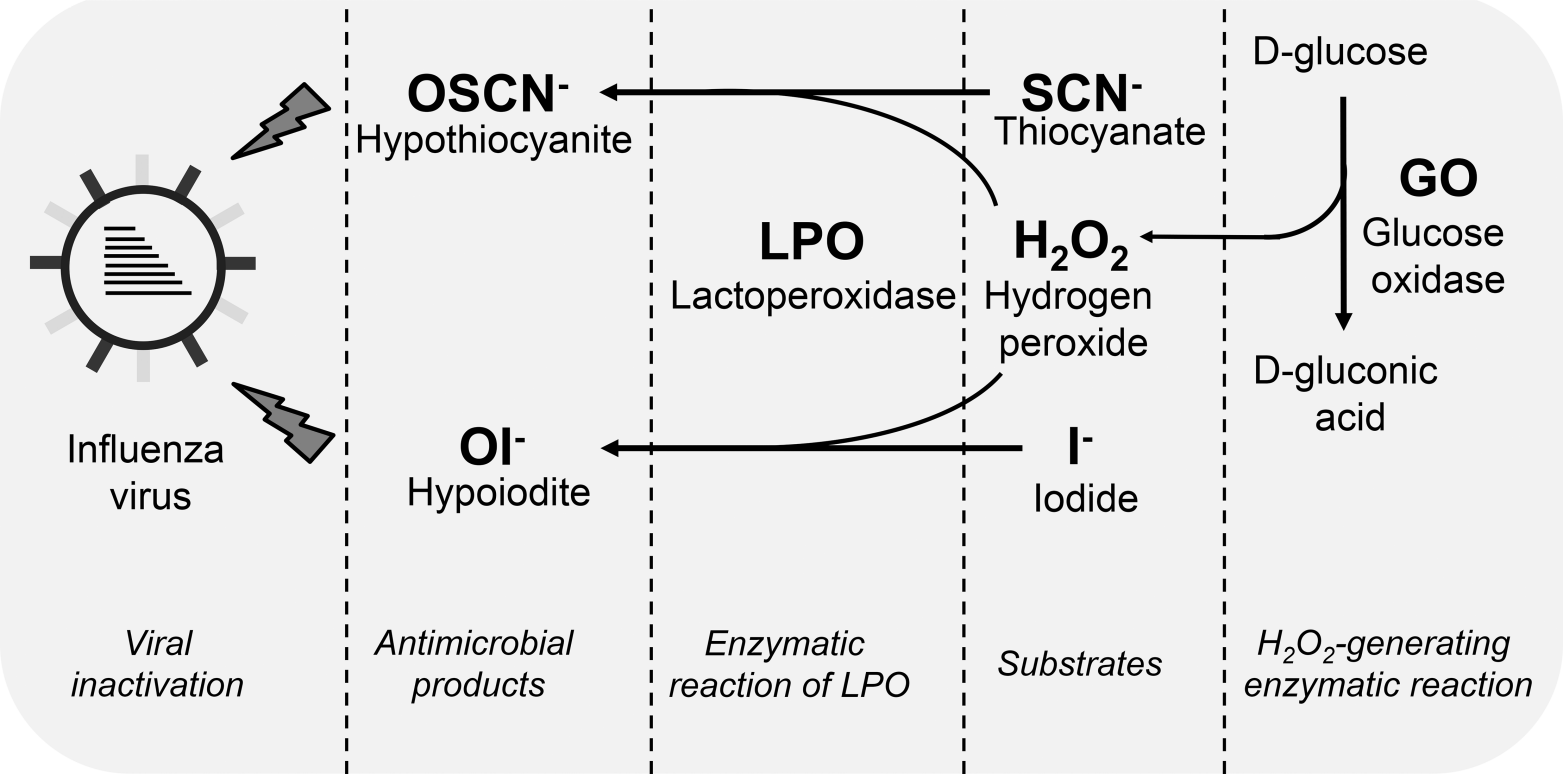
Description of the cell-free *in vitro* experimental system producing antiviral hypothiocyanite or hypoiodite. We established an *in vitro* experimental system to study the antiviral action of the LPO/H_2_O_2_/(SCN^-^/I^-^) system in the absence of epithelial cells. H_2_O_2_ is generated in the enzymatic reaction of GO turning D-glucose into D-gluconate. Produced H_2_O_2_ is used by LPO to oxidize its potential substrates, SCN^-^ or I^-^. The products of the enzymatic action of LPO are either OSCN^-^ or OI^-^, depending on the substrate used. Both, OSCN^-^ or OI^-^ have virucidal effects on influenza viruses. Physiologically relevant concentrations of LPO (6.5 μg/ml), SCN^-^ or I^-^ (400 μM), glucose (5 mM) and glucose oxidase (0.01 U/ml) are used. SCN^-^, thiocyanate; OSCN^-^, hypothiocyanite; I^-^, iodide; OI^-^, hypoiodite; LPO, lactoperoxidase; GO, glucose oxidase.

Bronchial epithelial cells (BEC) are a primary target for influenza virus replication [6]. Early response by BECs to influenza virus is crucial in determining progression of viral infection, adaptive immunity, and lung pathogenesis [7]. BECs orchestrate an oxidative extracellular antimicrobial system present in the airway surface liquid consisting of LPO, its main natural substrate, the thiocyanate anion (SCN^-^) and hydrogen peroxide (H_2_O_2_) (Fig 1) [8–11]. LPO is an abundant peroxidase in the airways that uses H_2_O_2_ to oxidize its most abundant and preferred substrate, SCN^-^, into hypothiocyanite (OSCN^-^) [12]. Hypothiocyanite is a short-lived, antimicrobial agent that is not toxic to the host and has been shown to kill several microbes, mainly bacteria. Its antiviral action remains, however, less characterized and understood.

Hypothiocyanite was to shown to inactivate the A/H1N1 2009 pandemic influenza virus in a cell-free system [13]. We also reported that OSCN^-^ generated on the apical surface of BECs is capable of inactivating the A/swine/Illinois/02860/09 (swH1N2) influenza A strain [9]. These observations suggest that the H_2_O_2_/LPO/SCN^-^ system is an important contributor to anti-influenza viral defense of the respiratory immune system, and imply that this innate response could be utilized to enhance early clearance of influenza virus perhaps in a strain-independent manner. To better understand the anti-influenza virus action of this oxidative system, we aimed at characterizing its virus-inactivating effect by testing several influenza virus strains. In addition to its natural substrate, SCN^-^, we also tested iodide (I^-^), as LPO substrate to study the substrate specificity of the antiviral action of LPO. LPO readily oxidizes several halides and pseudohalides using H_2_O_2_ [14]. Iodide is considered to be a better LPO substrate than SCN^-^ to inhibit growth of certain fungi and bacteria [15, 16]. In this study, we tested an *in vitro*, cell-free experimental system to characterize the anti-influenza virus action and substrate specificity of LPO. We found that LPO inactivates a wide-range of influenza strains, but surprisingly the extent of LPO-mediated viral inactivation and LPO substrate preference differed among tested isolates.

## Materials and methods

### Influenza A and B strains

Influenza A and B strains used in this work were purchased from the NIH Biodefense and Emerging Infections Research Resources Repository, NIAID, NIH (BEI Resources) and are listed in Table 1. Influenza viruses were propagated in Madin-Darby Canine Kidney (MDCK) cells (ATCC® CCL-34™) as described [9]. Briefly, MDCK cells were cultured in Dulbecco's modified Eagle's medium (DMEM) containing high glucose (HyClone) supplemented with 5% heat-inactivated fetal bovine serum (FBS; HyClone) and maintained at 37°C with 5% CO_2_. Viral strains were cultured in MDCK cells using infection medium (DMEM containing high glucose supplemented with 1 mM L-glutamine with 1-μg/ml tosylsulfonyl phenylalanyl chloromethyl ketone [TPCK]-treated trypsin). Viruses were collected 24–48 hours post-infection.

**Table 1:** Influenza virus strains used in this work.

Viral strain	Viral species	Serotype/subtype
**A/Brisbane/59/2007**	IAV	H1N1
**A/California/04/2009**	IAV	H1N1
**A/Mississippi/3/2001**	IAV	H1N1
**A/Turkey/Kansas/4880/1980**	IAV	H1N1
**A/Swine/Illinois/02860/2009**	IAV	H1N2
**A/Texas/50/2012**	IAV	H3N2
**A/Wisconsin/67/2005**	IAV	H3N2
**A/Aichi/2/1968**	IAV	H3N2
**A/Hong Kong/8/1968**	IAV	H3N2
**B/Yamagata/16/1988**	IBV	Yamagata
**B/Great Lakes/1739/1954**	IBV	Yamagata
**B/New York/1056/2003**	IBV	Victoria

IAV, Influenza A virus; IBV, Influenza B virus. Viruses were purchased from BEI Resources.

### Cell-free viral inactivation assay

Components of the LPO-based antiviral system were used at the following concentrations: 6.5 μg/ml LPO, 400 μM SCN^-^/I^-^, glucose (0.005 M) and glucose oxidase 0.01 U/mL. The reaction volume was set to 40 μL with the appropriate concentration of each component mentioned above. Catalase (700 U/mL, Sigma-Aldrich, St. Louis, MO) was also used when indicated to inhibit the system. The components were assembled in a sterile Eppendorf tube with the virus being added last. The tubes were then placed on a 37°C heating block for one hour. After the incubation, supernatants were stored at -80°C. Plaque assays were performed on MDCK cells to determine viral concentrations as previously described [9, 17]. This assay is the “cell-free” version of the viral inactivation assay established previously on primary airway epithelial cells with the main difference that the source of H_2_O_2_ here is the glucose/GO enzymatic reaction compared to Dual oxidase 1 in epithelial cells [9].

### Statistics

Data for the viral inactivation assay were log_10_-transformed and significance was calculated using a one-way ANOVA followed by Tukey or Dunn’s multiple comparison post-hoc test when more than two samples were compared. When virus inactivation or the substrate preference ratios were compared between two LPO substrates or influenza subtypes or species, Mann-Whitney test was used. Statistical analysis was performed using Prism 6 for Windows version 6.07 software. *, p<0.05; **, p<0.01; ***, p<0.001.

## Results

### Optimization of the cell-free, in vitro H_2_O_2_/LPO/(SCN^-^/I^-^) experimental system

LPO is produced by submucosal glands and epithelial cells in the respiratory tract and it accumulates in the airway surface liquid [18, 19]. LPO concentration in airway secretions has been estimated to be in the range of 3–12 μg/ml [18]. We used a physiologically relevant LPO concentration of 6.5 μg/ml in our cell-free assay [20]. Thiocyanate is present in airway secretions in submillimolar concentrations [12, 18]. Accordingly, we used 400 μM SCN^-^ in the *in vitro* assay, and used 400 μM iodide as alternative LPO substrate. The same LPO and SCN^-^ concentrations were used as previously published in our prior studies using primary bronchial epithelial cells to make results comparable between the cell-free system and epithelial cultures [9]. While H_2_O_2_ has been detected in airway secretions of humans and other mammalian species, estimating its basal levels is difficult due to its volatile nature. The main *in vivo* sources of H_2_O_2_ in the airways are NADPH oxidases called Dual Oxidase 1 and 2 (Duox 1/2) [8]. Duox enzymes are highly expressed in the apical plasma membrane of BECs and generate H_2_O_2_ directly into the airway surface liquid [8]. In our *in vitro* system containing BECs, Duox enzymes generate H_2_O_2_ driving the antimicrobial action of LPO [9, 20, 21]. In the cell-free system described here, H_2_O_2_ is provided by the enzymatic reaction of glucose (5 mM) and glucose oxidase (GO) that produces D-gluconic acid as the end product (Fig 1) [20]. Generating H_2_O_2_ by an enzymatic reaction better models the slow but maintained nature and kinetics of H_2_O_2_ release by BECs than a bolus-like addition of H_2_O_2_ [20, 21]. To mimic the H_2_O_2_ output of BECs in the cell-free system, we titrated the dose of GO and chose 0.01 U/ml, a concentration that is the closest to the hourly H_2_O_2_ production of BECs [9, 20, 21] (data not shown). Thus, the components of the cell-free system are used in concentrations that are physiologically relevant.

### The cell-free H_2_O_2_/LPO/(SCN^-^/I^-^) system inactivates the A/Swine/Illinois/02860/2009 H1N2 influenza virus

**Fig 2:**
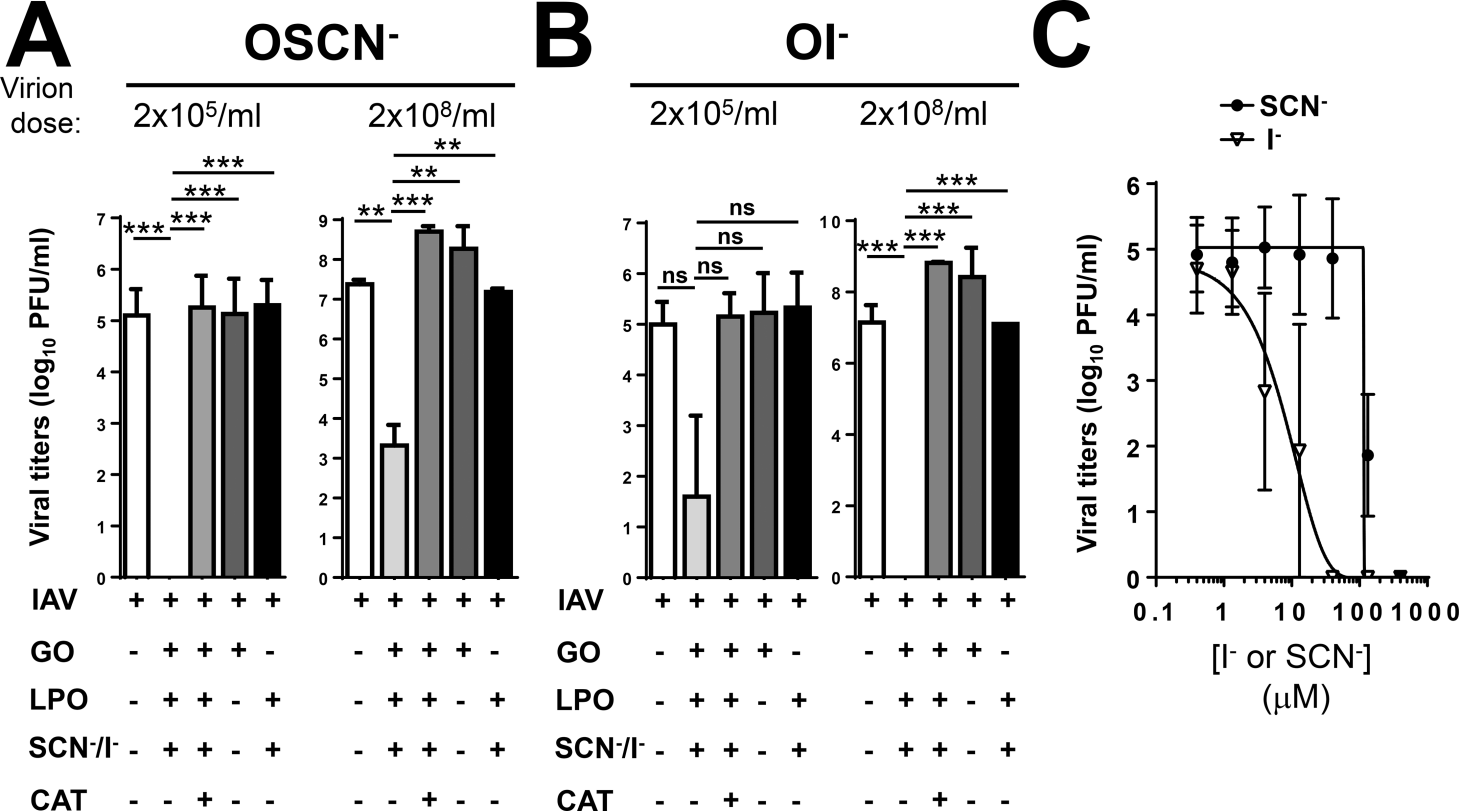
The cell-free H_2_O_2_/LPO/(SCN^-^/I^-^) system inactivates A/Swine/Illinois/02860/2009 H1N2 influenza A virus. The antiviral action of the cell-free H_2_O_2_/LPO/(SCN^-^/I^-^) system was tested against the A/Swine/Illinois/02860/2009 (H1N2) influenza A virus. Viruses were incubated in the presence or absence of the components of the cell-free system as indicated for 1 hour when (A) SCN^-^ or (B) I^-^ was used as LPO substrate. Viral inactivation was assessed by plate-forming unit assay using MDCK cells. Mean+/-S.E.M., n = 4–5. (C) SCN^-^ and I^-^ dose-dependence of A/Swine/Illinois/02860/2009 inactivation. Mean+/-S.E.M., n = 3. One-way ANOVA, Tukey’ multiple comparison test. Ns, non-significant, **, p<0.01; ***, p<0.001. SCN^-^, thiocyanate; OSCN^-^, hypothiocyanite; I^-^, iodide; OI^-^, hypoiodite; LPO, lactoperoxidase; GO, glucose oxidase; MDCK, Madin-Darby canine kidney cells; PFU, plaque-forming unit.

We have shown previously that BECs are capable of inactivating the A/Swine/Illinois/02860/2009 (H1N2) influenza A strain (Table 1) in a H_2_O_2_ (Duox)-, LPO- and SCN^—^dependent manner [9]. Next we wanted to confirm this in the cell-free system. As shown in Fig 2A, the H_2_O_2_/LPO/SCN^-^ cell-free system had also a strong and substantial inactivating effect on the A/Swine/Illinois/02860/2009 (H1N2) strain. Importantly, viral inactivation in the cell-free system was entirely blocked by the addition of catalase, a H_2_O_2_-scavanging enzyme (Fig 2A). No viral inactivation was observed when only GO was added but LPO and SCN^-^ were omitted from the system (Fig 2A). Similarly, having LPO and SCN^-^ without the H_2_O_2_-generating GO had no substantial viral-inactivating effect (Fig 2A). Comparable data were observed on BECs [9] indicating that H_2_O_2_ alone is inefficient and OSCN^-^ is responsible for inactivating the H1N2 influenza strain in both systems.

Next, we tested how H1N2 inactivation by the cell-free system changes if I^-^ (an alternative LPO substrate) is used. I^-^ has been shown to enhance the antiviral activity of LPO against adenovirus and respiratory syncytial viruses [15, 22]. I^-^ was used at the same concentration as SCN^-^ (400 μM). Replacing SCN^-^ with I^-^ in the cell-free system led to even more effective H1N2 inactivation (Fig 2A). Similar to results obtained with SCN^-^, catalase blocked viral inactivation when I^-^ was used (Fig 2B). Omitting any of the components of the I^—^based cell-free system resulted in complete loss of H1N2 inactivation confirming that hypoiodite (OI^-^) is responsible for the virucidal effect (Fig 2B). To better characterize the substrate preference of LPO in inactivating H1N2, we tested viral inactivation at doses of SCN^-^ and I^-^ lower than 400 μM. As the dose-dependence curves shown in Fig 2C indicate, H1N2 was more susceptible to I^—^mediated inactivation by the cell-free system. Interestingly, sensitivity to OSCN^-^ drops drastically at SCN^-^ doses just below 100 μM (Fig 2C). Results presented here confirm that the cell-free system serves as a good model to study influenza inactivation by the H_2_O_2_/LPO/SCN^-^ system, and the swine H1N2 strain tested is susceptible to both, OSCN^-^ and OI^—^mediated inactivation by LPO, with a slightly higher sensitivity to OI^-^.

### H1N1 influenza A strains are inactivated by LPO in a substrate-independent manner

**Fig 3:**
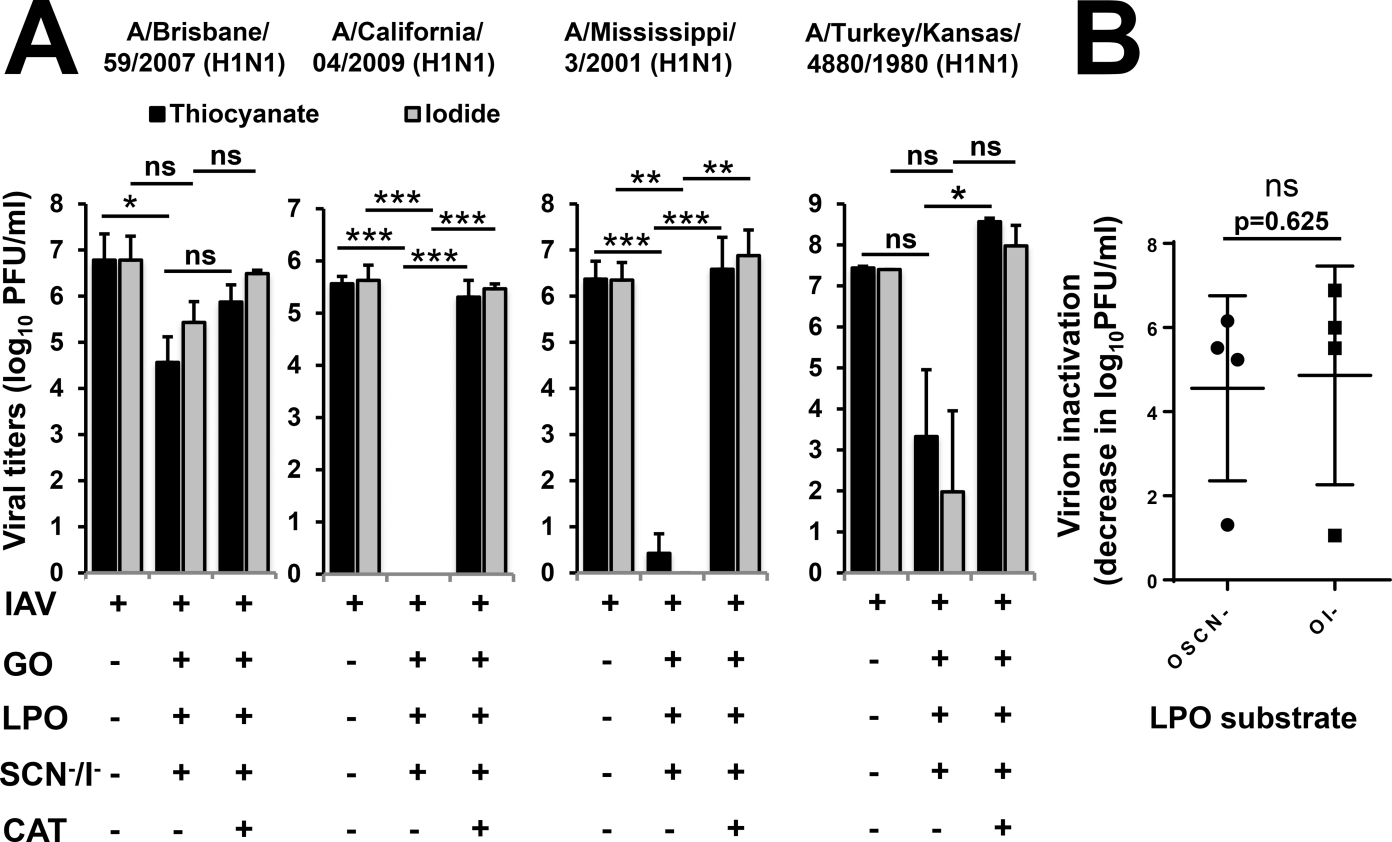
Hypoiodite and hypothiocyanite are equally efficient in inactivating H1N1 influenza A viruses in the cell-free system. (A) The antiviral action of the cell-free H_2_O_2_/LPO/(SCN^-^/I^-^) system was tested against H1N1 influenza A strains, A/Brisbane/59/2007 (n = 3), A/California/04/2009 (n = 5), A/Mississippi/3/2001 (n = 4) and A/Turkey/Kansas/4880/1980 (n = 2). Viruses were incubated in the presence or absence of the components of the cell-free system as indicated for 1 hour and viral inactivation was assessed by plate-forming unit assay using MDCK cells. Mean+/-S.E.M. One-way ANOVA, Dunn’s multiple comparison test. (B) No significant difference can be observed in virus inactivation of the four H1N1 strains tested. Virus inactivation is calculated as the difference in viable viral titers between the sample containing the cell-free system and the sample containing the full, cell-free system plus catalase. Mean+/-S.E.M., n = 2–5. Mann-Whitney test. Ns, non-significant, *, p<0.05; **, p<0.01; ***, p<0.001. SCN^-^, thiocyanate; OSCN^-^, hypothiocyanite; I^-^, iodide; OI^-^, hypoiodite; LPO, lactoperoxidase; GO, glucose oxidase; MDCK, Madin-Darby canine kidney cells; PFU, plaque-forming unit; IAV, Influenza A virus.

Influenza A viruses are classified based on two of their surface proteins, hemagglutinin (HA) and neuraminidase (NA), that also determine their antigenic specificities. Since the H1N1 and H3N2 serotypes have been commonly present in human epidemics and pandemics over the last century [23], it was important to compare several H1N1 and H3N2 strains in the cell-free system for LPO-mediated inactivation (Table 1). The IAV H1N1 strains, A/Brisbane/59/2007, A/California/04/2009 and A/Mississippi/3/2001 (Table 1), cause seasonal influenza virus infections in humans while the A/Turkey/Kansas/4880/1980 H1N1 strain is a virus of swine antigenic phenotype isolated from turkeys [23–25]. We tested A/Brisbane/59/2007 and observed a 1.31+/-0.42 (mean+/-S.E.M., n = 3) log (SCN^-^) and a 1.06+/-0.28 (mean+/-S.E.M., n = 3) log (I^-^) inactivation by the cell-free system, respectively (Fig 3A). The extent of OSCN^—^mediated viral inactivation was significant (p = 0.011) while that of OI^-^ was not (p = 0.090) (Fig 3A). Catalase had an inhibitory but non-significant (p = 0.092) and incomplete effect on viral inactivation for both substrates (Fig 3A). Both LPO substrates mediated A/California/04/2009 viral inactivation beyond detection limit of the assay in the cell-free system which was highly significant (p<0.0001, for each substrate) (Fig 3A). Removing H_2_O_2_ by catalase treatment significantly reversed (p<0.0001) the virus-inactivating effect of LPO by both substrates (Fig 3A). The A/Mississippi/3/2001 strain was also efficiently inactivated by LPO in a substrate-independent manner (log-based extent of inactivation: 6.16+/-0.27 for OSCN^-^ while 6.88+/-0.55 for OI^-^, n = 4) that was entirely blocked by catalase (Fig 3A). Although the A/Turkey/Kansas/4880/1980 H1N1 strain was also efficiently inactivated by LPO, its extent did not reach levels of significance (Fig 3A). While the four H1N1 influenza strains tested did not show a strong substrate preference in terms of inactivation by the cell-free H_2_O_2_/LPO/(SCN^-^/I^-^) system, they differed in their susceptibilities (Fig 3A). This is also shown in Fig 3B where the extent of virus inactivation is calculated as the catalase-dependent difference in viable viral titers (viral doses detected in presence of the full cell-free system are subtracted from viral doses recovered in presence of the full cell-free system plus catalase). Virus inactivation is calculated throughout the entire manuscript the same way.

### Iodide is the preferred LPO substrate to inactivate H3N2 IAV strains

**Fig 4:**
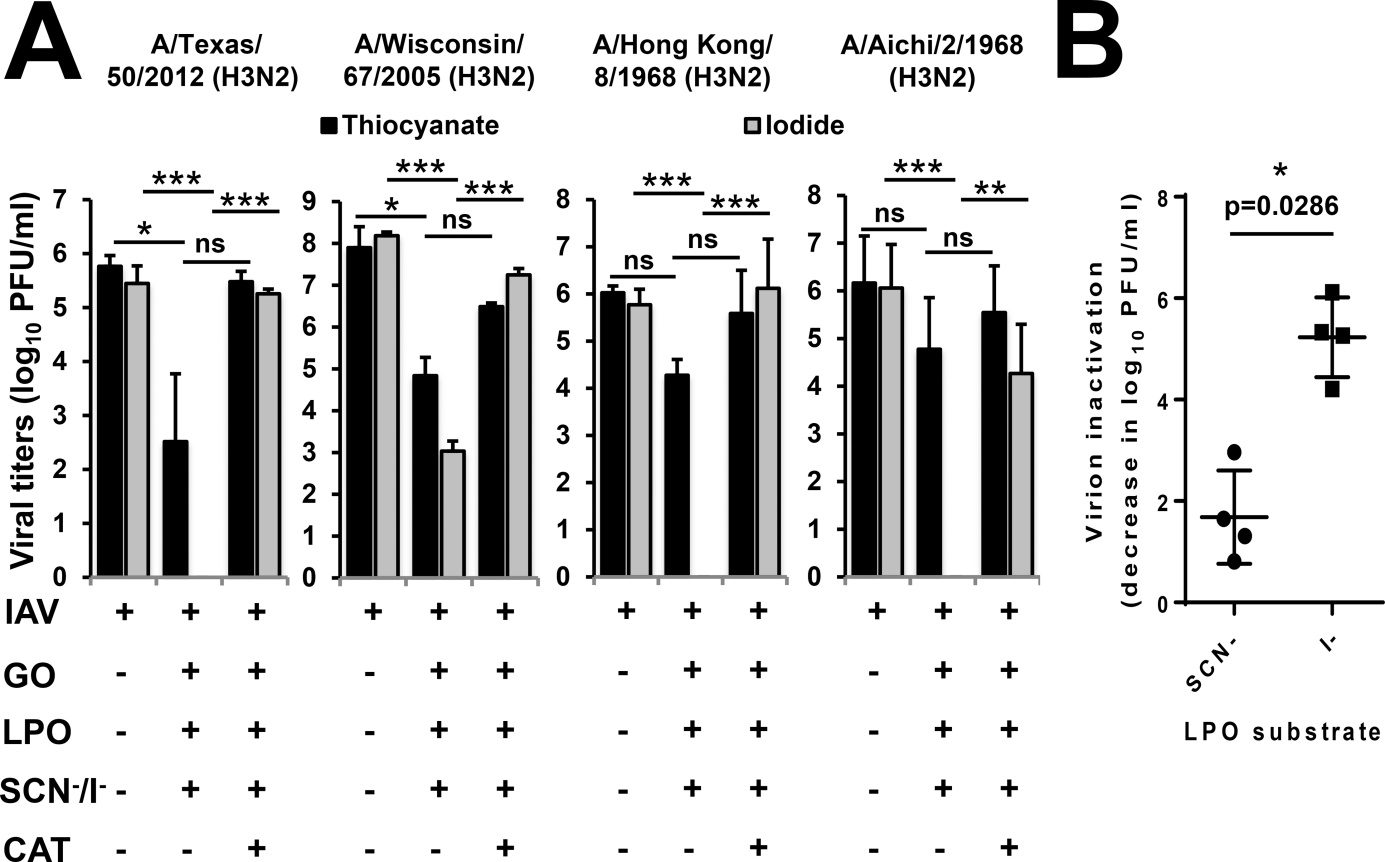
Hypoiodite is more efficient in inactivating H3N2 influenza A viruses than hypothiocyanite in the cell-free system. (A) The antiviral action of the cell-free H_2_O_2_/LPO/(SCN^-^/I^-^) system was tested against H3N2 influenza A strains: A/Texas/50/2012 (n = 3), A/Wisconsin/67/2005 (n = 2), A/Hong Kong/8/68 (n = 4) and A/Aichi/2/68 (n = 5). Viruses were incubated in the presence or absence of the components of the cell-free system as indicated for 1 hour and number of viable viruses was assessed by plate-forming unit assay using MDCK cells. Mean+/-S.E.M. One-way ANOVA, Dunn’s multiple comparison test. (B) Virus inactivations of the H3N2 strains are compared according to the LPO substrates used. Mean+/-S.E.M., n = 2–5. Mann-Whitney test. IAV, Influenza A virus; SCN^-^, thiocyanate; I^-^, iodide; LPO, lactoperoxidase; GO, glucose oxidase; MDCK, Madin-Darby canine kidney cells; PFU, plaque-forming unit.

H3N2 IAV strains have become dominant causes of recent seasonal influenza epidemics [1]. The following four H3N2 influenza A strains were tested in the cell-free system for their LPO substrate specificities: A/Texas/50/2012, A/Wisconsin/67/2005, A/Aichi/2/1968 and A/Hong Kong/8/1968 (Table 1). These H3N2 strains circulate in the human population causing seasonal infections [26–29]. As shown in Fig 4A, OSCN^-^ treatment resulted in a decrease of infectious virus titers as measured by plaque assay between 1–3 logs for these H3N2 strains. While OSCN^—^mediated inactivation of A/Texas/50/2012 (p = 0.049) and A/Wisconsin/67/2005 (p = 0.023) were significant, there were no detectable significant differences for A/Aichi/2/68 (p = 0.155) and A/Hong Kong/8/68 (p = 0.123) (Fig 4A). Catalase reversed the H3N2 virus-inactivating effects of OSCN^-^ (Fig 4A). When OSCN^-^ was replaced by I^-^ as the LPO substrate in the cell-free system, it robustly enhanced viral inactivation that was significant (p<0.0005) for all four H3N2 IAVs (Fig 4A). The formation of OI^-^ by LPO resulted in reduced titers of A/Wisconsin/67/2005 (4.21+/-0.25 log decrease, mean+/-S.E.M., n = 3), all other H3N2 strains were completely eliminated by the treatment (Fig 4A). The reversal of OI^—^mediated H3N2 inactivation by catalase was highly significant (p<0.01) with all four strains tested (Fig 4A). We determined H3N2 IAV inactivation in the cell-free system and found it to be significantly (p = 0.029) more robust when I^-^ was the LPO substrate used, compared to SCN^-^ (Fig 4B).

**Fig 5:**
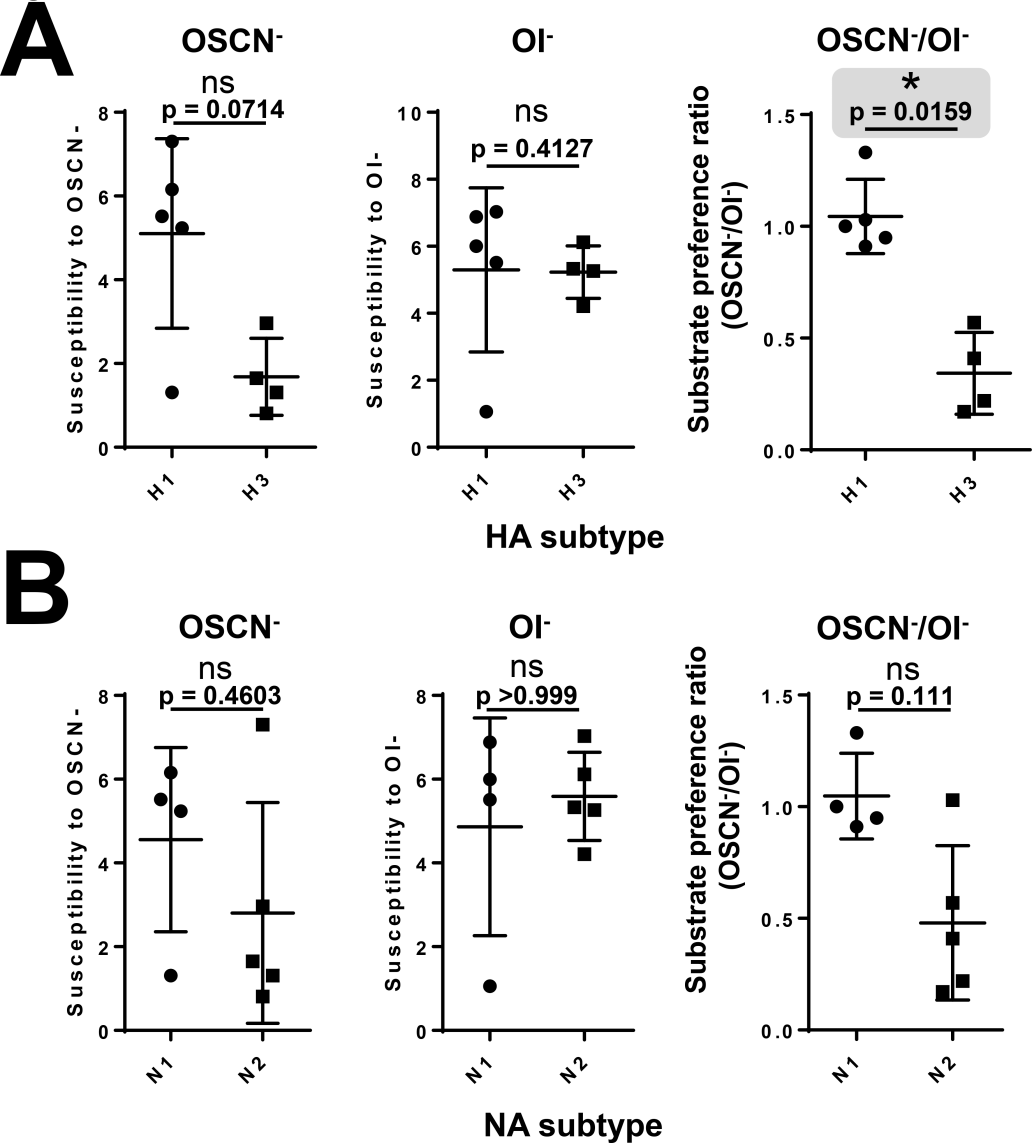
Hemagglutinin subtypes are associated with LPO substrate preference supporting IAV inactivation. Susceptibilities of tested IAV strains to OSCN^-^ and OI^-^ in the cell-free system were compared according to their types of (A) hemagglutinin (H1, H3) and (B) neuraminidase (N1, N2). “SCN^-^/I^-^ substrate preference ratios” were also calculated as described in the text for all nine IAV strains and compared among HA and NA types (upper and lower right panels). Ns, non-significant, *, p<0.05. SCN^-^, thiocyanate; OSCN^-^, hypothiocyanite; I^-^, iodide; OI^-^, hypoiodite; LPO, lactoperoxidase; GO, glucose oxidase; HA, hemagglutinin; NA, neuraminidase. The gray area highlights the only significant difference in the figure.

To assess potential association of hemagglutinin or neuraminidase types of IAVs with their susceptibility to the LPO-based system, we compared viral inactivation and substrate preference of all nine IAV strains tested. When IAVs tested so far were grouped according to their HA types (H1 and H3) or NA types (N1, N2), no significant differences were seen between them regarding OSCN^—^or OI^—^mediated viral inactivation (Fig 5A and 5B, left and middle panels). To better characterize the substrate preference of LPO-mediated viral inactivation, we calculated a “SCN^-^ / I^-^ substrate preference ratio” or “OSCN^-^/OI^-^ susceptibility ratio” for each strain by dividing the extent of SCN^—^fueled virus inactivation by the extent of the I^—^mediated one. When these SCN^-^/I^-^ substrate preference ratios of all nine IAV strains were grouped according to their HA type (irrespective of NA), they were significantly (p = 0.0159) higher with H1 compared to H3 (Fig 5A, right panel). When the substrate preference ratios were grouped according to the NA type (irrespective of HA), we found no significant (p = 0.111) difference (Fig 5B, right panel). In summary, H3N2 IAV strains are also susceptible to the antimicrobial actions of LPO and show a surprising preference for I^-^ as the preferred substrate for inactivation that is associated with H1 and H3 hemagglutinin subtypes.

### The influenza B virus strain, B/Yamagata/16/1988, is more susceptible to hypothiocyanite than hypoiodite

**Fig 6:**
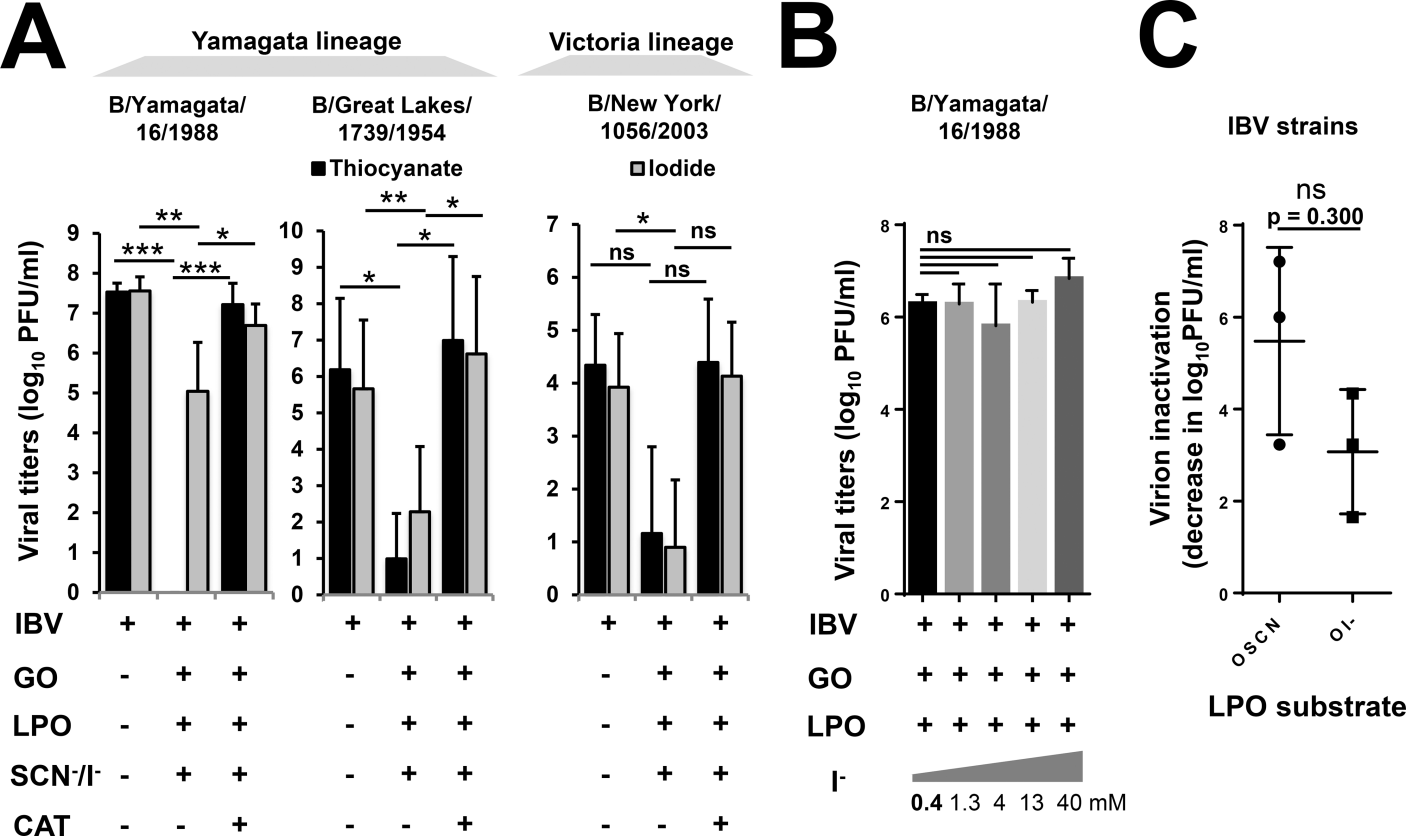
LPO substrate preference of Influenza B virus inactivation is strain-dependent. (A) The antiviral action of the cell-free H_2_O_2_/LPO/(SCN^-^/I^-^) system was tested against influenza B strains: B/Yamagata/16/1988, B/Great Lakes/1739/1954 and B/New York/1056/2003. Viruses were incubated in the presence or absence of the components of the cell-free system as indicated for 1 hour and viral inactivation was assessed by plate-forming unit assay using MDCK cells. Mean+/-S.E.M., n = 5. One-way ANOVA, Dunn’s multiple comparison test. (B) B/Yamagata/16/1988 Virus inactivation was measured at increasing I^-^ concentrations (0.4–40 mM) in the cell-free system by the PFU assay. Mean+/-S.E.M., n = 5. One-way ANOVA, Dunn’s multiple comparison test. (C) The extents of IBV inactivation by OSCN^-^ and OI^-^ of the three strains tested were compared. Mean+/-S.E.M., Mann-Whitney test. Ns, not significant; *, p<0.05; **, p<0.01; ***, p<0.001. SCN^-^, thiocyanate; OSCN^-^, hypothiocyanite; I^-^, iodide; OI^-^, hypoiodite; LPO, lactoperoxidase; GO, glucose oxidase; MDCK, Madin-Darby canine kidney cells; PFU, plaque-forming unit; IBV, Influenza B virus.

IBV strains primarily infect humans, do not have established animal reservoirs and pose little risk of pandemic infection [30, 31]. IBV may cause seasonal influenza infections and co-circulate with IAV strains [30]. Importantly, IBV can dominate influenza seasons and is typically more resistant to antivirals than IAVs [30, 32]. We investigated whether LPO present in our cell-free system can inactivate the influenza B strain, B/Yamagata/16/1988, a prototype strain of the Yamagata lineage of IBV strains [33]. As shown in Fig 6A, OSCN^-^ led to complete, highly significant (p<0.001) inactivation of the viral dose used. Using I^-^ as the LPO substrate, viral inactivation of IBV was significant (p = 0.021), but was reduced to a 1.6+/-0.62 log decrease (n = 5) in viral titers compared to 7.2+/-0.27 log drop (n = 5) in viral titers following OSCN^-^ production (Fig 6A). As in the case of IAV strains, both, OSCN^—^and OI^—^mediated inactivation of this IBV strain was completely inhibited by catalase, as well (Fig 6A). Since the used dose of I^-^ sustained only minor inactivation of the tested IBV strain, we increased the I^-^ concentration to 40 mM to explore the potential improvement of the antiviral action of LPO. Unexpectedly, we did not observe improved inactivation of the B/Yamagata/16/1988 strain by up to 100-fold higher I^-^ doses (Fig 6B). Thus, the tested IBV strain showed a preference for SCN^-^ as the LPO substrate for inactivation.

### The substrate preference of influenza B strains for LPO-mediated inactivation is strain-dependent

To test whether the observed, marked difference in susceptibility to OSCN^-^ versus OI^-^ of the B/Yamagata/16/1988 strain (Fig 6A and 6B) is a unique feature of this strain or is generally true for all IBV strains, we tested two additional IBV strains. The B/Great Lakes/1739/1954 strain that also belongs to the Yamagata lineage [34] was efficiently inactivated (n = 5) by both, OSCN^-^ (6.00+/-0.98 log decrease) and OI^-^ (4.34+/-1.13 log decrease), with a slight preference to OSCN^-^ (Fig 6A). The B/New York/1056/2003 IBV strain belongs to the Victoria lineage and has been isolated from a pharyngeal swab from a human with unspecified respiratory disease (BEI Resources). This Victoria lineage strain was equally inactivated by OSCN^-^ (3.23+/-1.00 log decrease) and OI^-^ (3.23+/-0.75 log decrease, n = 3) (Fig 6A). LPO-mediated inactivation of all three IBV strains was entirely blocked by catalase (Fig 6A). Although there is a trend towards OSCN^-^, data shown in Fig 6C did not find an overall significant difference between OSCN^—^fueled and OI^—^mediated inactivation of IBV strains.

### Influenza B viruses have a significantly higher OSCN^-^/OI^-^ susceptibility ratio than influenza A strains

**Fig 7:**
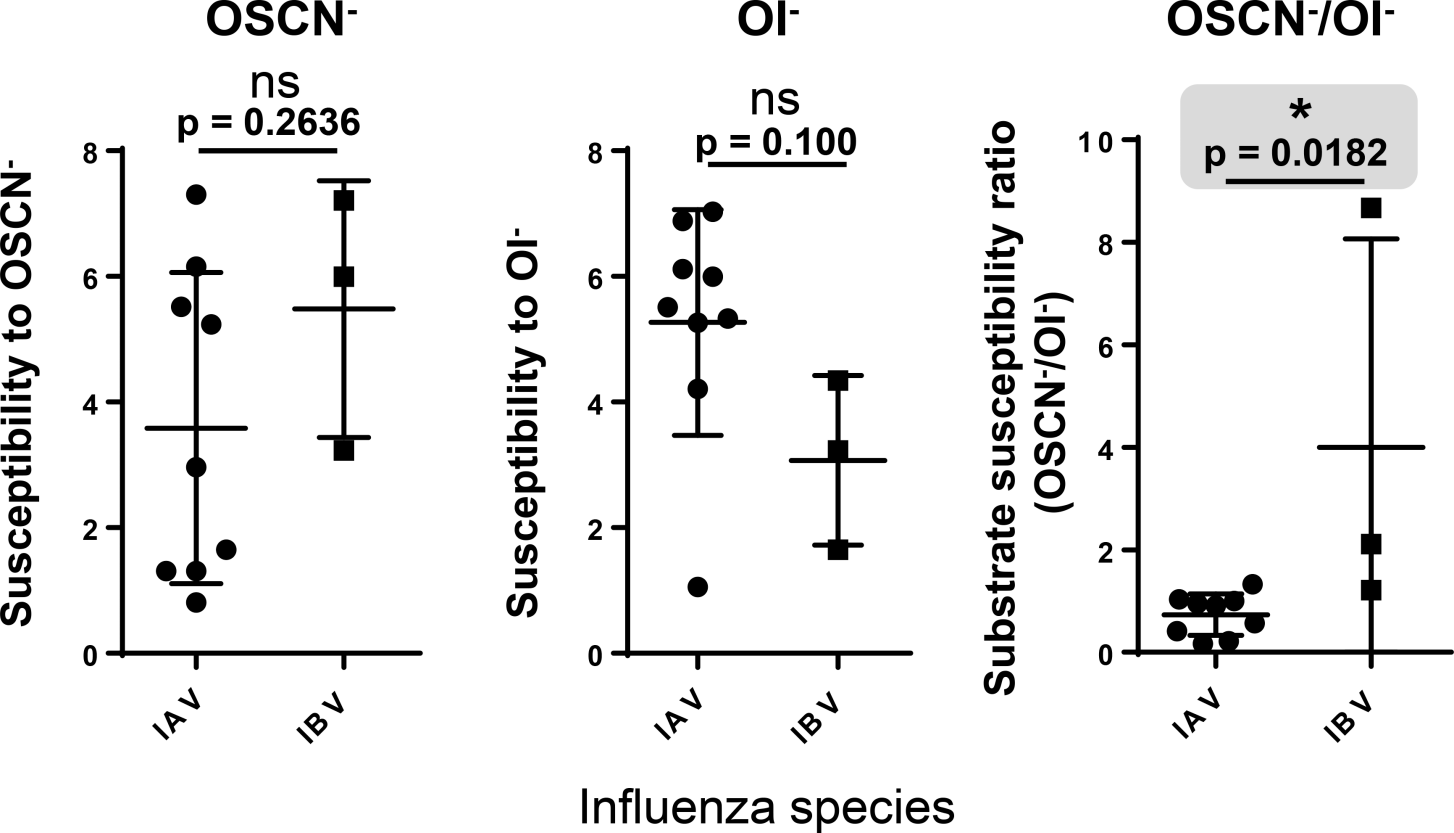
**Comparison of influenza A and B strains for their susceptibilities to the virucidal effects of LPO.** The nine IAV and three IBV influenza strains tested were compared regarding their susceptibilities to OSCN^-^ (left panel), to OI^-^ (middle panel) or their LPO substrate preference ratios (right panel) in the cell-free system. This figure does not show new experimental data but presents new analysis of experimental results obtained in Figs 2–6. The gray area highlights the only significant difference in the figure. Mean+/-S.E.M., Mann-Whitney test. Ns, not significant; *, p<0.05. OSCN^-^, hypothiocyanite; OI^-^, hypoiodite; IAV, influenza A virus; IBV, influenza B virus.

To explore whether there are differences in the previous readouts between IAV and IBV strains, we compared susceptibilities to OSCN^-^ and OI^-^, as well as, their ratio called “OSCN^-^/OI^-^ susceptibility ratio” between the IAV and IBV strains analyzed. As Fig 7 shows, there was no significant difference between the two viral species in their susceptibilities to the virucidal agents. However, we observed a significant difference (p = 0.0182) in their susceptibility ratios as shown in Fig 7 (right panel). All IAV strains had very low ratios (0.73+/-0.13, mean+/-S.E.M., n = 9) while IBV strains had much higher values (4.00+/-2.35, mean+/-S.E.M., n = 3) (Fig 7). Thus, IBV strains tend to be more sensitive to OSCN^-^ whereas IAV strains are equally sensitive to both LPO substrates or more susceptible to OI^-^.

### Substrate specificity map of influenza inactivation by LPO

**Fig 8:**
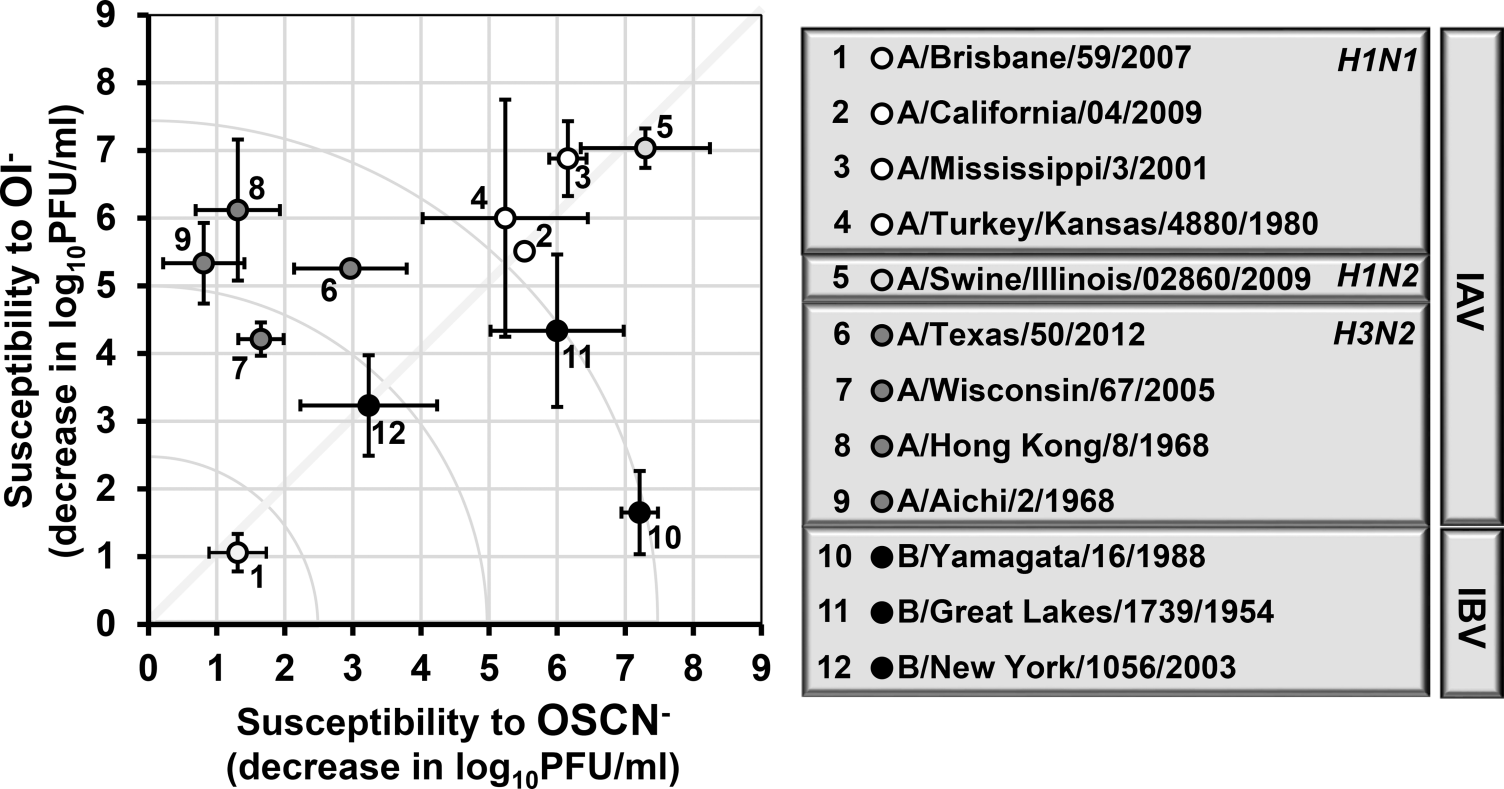
LPO substrate specificity and susceptibility map of influenza strains. Susceptibilities of all twelve influenza strains tested in this study against OSCN^-^ or OI^-^ in the cell-free system are plotted. Names, species, serotypes or subtypes of the viral strains are indicated. Susceptibility is defined as the decrease in viral doses (log_10_PFU/ml) in the cell-free system following catalase treatment—see text for further details. The X axis shows susceptibility against OSCN^-^ while the Y axis shows susceptibility towards OI^-^. Mean+/-S.E.M. for both, X and Y axes, n = 2–5. The indicated diagonal crossing on the map from the lower left corner to the upper right corner indicates no substrate preference, irrespective of susceptibility. The dotted line named as the “H3N2 cluster” indicates that all four H3N2 strains group close to each other. The quadrant circles indicate viral inactivations of different sizes (2.5, 5.0 and 7.5 logs). OSCN^-^, hypothiocyanite; OI^-^, hypoiodite; LPO, lactoperoxidase; PFU, plaque-forming unit; IAV, Influenza A virus; IBV, Influenza B virus.

To summarize our results after surveying 12 different influenza strains for their susceptibilities and LPO substrate preferences in the H_2_O_2_/LPO/(SCN^-^/I^-^) system, we plotted our data as a “substrate preference or susceptibility map” shown in Fig 8. The X-axis represents the extent of viral inactivation that is associated with OSCN^-^, while the Y-axis represents the extent of viral inactivation by OI^-^. The higher the number, the more susceptible a particular virus strain is to the indicated, LPO-generated antiviral agent (Fig 8). The indicated diagonal crossing the map from the lower left corner to the upper right corner indicates no substrate preference (Fig 8). The H1N2 influenza strain tested localizes to the upper right corner indicating high susceptibility to the LPO-based antimicrobial system with no considerable preference for the LPO substrate (Fig 8). All H1N1 strains appear on the diagonal showing no preference for the LPO substrate. While the A/Brisbane/59/2007 strain can be found in the lower left corner, the A/California/04/2009, A/Mississippi/3/2001, and A/Turkey/Kansas/4880/1980 strains are, however, all located in the upper right corner, close to the H1N2 strain, representing widely distinct susceptibilities of the tested H1N1 viruses towards the H_2_O_2_/LPO/(SCN^-^/I^-^) system. All four H3N2 influenza A strains showed a clear and noteworthy preference for I^-^ as the LPO substrate for their inactivation in the cell-free system and were grouped together into an “H3N2 cluster” (Fig 8). The first IBV strain tested (B/Yamagata/16/1988) mapped into the lower right corner indicating its highly susceptible nature to OSCN^-^ but almost resistance towards OI^-^ (Fig 8). Two further IBV strains, however, failed to confirm this trend and localized to the vicinity of the center of the graph. Overall, our work represents an unexpected complexity of influenza virus inactivation by the H_2_O_2_/LPO/(SCN^-^/I^-^) system.

## Discussion

Seasonal and pandemic infections by influenza viruses represent a major threat. Seasonal influenza viruses infect 5–15% of the human population annually, resulting in more than half a million deaths worldwide [35]. Strain complexity, virus drift, and viral reassortment make it difficult to develop vaccines providing protection against a wide range of influenza strains. Current trivalent vaccines include one H1N1, one H3N2 IAV strain and one IBV strain of either the Yamagata or Victoria lineages [35]. Quadrivalent vaccines include two influenza B strains, one Yamagata and one Victoria strain, in addition to the two IAV strains [35]. The efficacy of the annual influenza vaccine varies widely from 10–60% [36], and the vaccine does not provide universal influenza virus protection [36]. Currently, all antivirals face the problem of increasing drug resistance [37]. Therefore, there is an urgent need to find novel mechanisms by which influenza can be fought, preferably in a strain-independent fashion.

The H_2_O_2_/LPO/(SCN^-^/I^-^) system represents an oxidative, fast-reacting mechanism of the respiratory innate immune system that is capable of killing or inactivating a wide range of pathogens. Our results reported here are one of the first ones to document its efficacy against influenza virus. Our prior results established that the H_2_O_2_/LPO/SCN^-^ system assembled in *in vitro* cultures of differentiated, human and rat, tracheobronchial epithelial cells inactivates the A/Swine/Illinois/02860/2009 influenza A strain [9]. Here we confirmed that H_2_O_2_/LPO/SCN^-^ system behaves in a very similar fashion and inactivates the same H1N2 strain under cell-free experimental conditions. This cell-free system provides us with an important tool to address the specificity and the mechanism of action of the LPO-based system against influenza viruses. It is important to emphasize that influenza viruses are inactivated by the H_2_O_2_/LPO/(SCN^-^/I^-^) system in both the cell-free and airway epithelial model [9] in less than 60 minutes. Very few, if any, mechanism of the innate immune system reacts so fast and efficiently.

In the current work we expanded our observation made on the IAV H1N2 strain to other influenza strains. Our central focus has been to address the question of whether SCN^-^ or I^-^ serves as a better substrate for the anti-influenza action of LPO. We revealed a LPO substrate preference of influenza inactivation that appears more complex than expected. Based on the current survey of influenza strains we conclude that H3N2 IAV strains are inactivated by OI^-^ more effectively than by OSCN^-^. This is presented as an “H3N2 cluster” on LPO’s substrate preference map (Fig 8). We did not find this bias towards I^-^ in case of the other IAVs (H1N1 and H1N2) that were inactivated by the LPO substrates. The potential explanation for this is unclear at the moment but redox-sensitive disulfide bonds may be different among HA or NA subtypes that could help explain this observation [38, 39]. Differences in OI^—^or OSCN^—^mediated oxidative modifications of these bonds could lead to altered virion disassembly or inhibition of viral entry into host cells [39, 40]. Oxidative stress has been shown to inactivate influenza viruses [41]. Oxidative damage of the lipid components of the viral envelope or the nucleoprotein could be also the results of the antimicrobial action of LPO [41]. Differences in susceptibilities of influenza viruses to polyphenols were linked to viral envelope glycoproteins [42]. OI^-^ has a higher oxidative strength than OSCN^-^ [43]. While the main potential targets of OSCN^-^/HOSCN are certain SH groups, OI^-^/HOI can target SH groups, NAD(P)H, reduced pyridine nucleotides, thioether groups and NH2 groups [43]. These molecular differences could form the basis for the observed inactivation patterns of influenza viruses in our study. The virucidal effect of OSCN^-^ and OI^-^ may be due to their ability to either interact with viral HA or NA directly and interfere with viral cell entry or to cross the outer layer of the viral envelop before oxidizing critical viral elements inside the virion. We know that beneath the lipid membrane of the influenza virus, a viral protein called M1 matrix protein is expressed and may be reacting with OSCN^-^ or OI^-^ and interfere with viral infectivity [44]. Other viral proteins that are cysteine-rich may be the targets of these antiviral agents. In bacteria, for example, the reported bacterial targets of OSCN^-^ are critical cysteine residues found in glycolytic enzymes, such as glyceraldehyde triphosphate dehydrogenase, hexokinase, glycose-6-phosphate dehydrogenase, and aldolase leading to the hypothesis that OSCN^-^ effect on bacterial growth is glycolysis-mediated [45]. Glucose transport and respiration may also be the target of OSCN^-^ and OI^-^. It has been reported to inhibit the activity of urease, which is critical to the ability of H. pylori to alkalinize gastric juice and colonize the stomach [46].

IBVs can cause symptoms similar to IAVs in humans, and are responsible for a large proportion of seasonal influenza infections [30, 32]. Far less is known regarding the viral and host determinants of IBV disease pathogenicity [30, 32]. The immune response against IBV is affected by yearly trivalent or quadrivalent vaccines and typically is weaker in humans than that against IAVs [30]. NA inhibitors that work well against most strains of IAV are typically less effective for IBVs [30]. Therefore, novel approaches are needed to confront IBV. Our results show that the B/Yamagata/16/1988 strain is sensitive against the H_2_O_2_/LPO/SCN^-^ system but remains more resistant against the H_2_O_2_/LPO/I^-^ system. After testing two other IBV strains, this pattern failed to be confirmed suggesting that it could be strain-specific. Testing a larger number of IBV strains is needed for a firmer conclusion. Nevertheless, our findings are the first to show that the H_2_O_2_/LPO/SCN^-^ antimicrobial system efficiently inactivates IBV strains, as well.

Given the wealth of historical data showing that these LPO-generated products have antimicrobial effects, they could be used for either therapeutic or prophylactic supplementation before infection or drugs, in combination with current anti-influenza therapies to enhance viral elimination and diminish inflammation. SCN^-^ is present in the diet and no serious adverse effects of SCN^-^ in humans have been reported at physiological concentrations (reviewed in [47]). Iodide intake has already been proposed to improve viral clearance but has several hurdles. While lower dose of iodide intake is safe and associated with normal thyroid function, recent works have proven that alterations in thyroid function are associated with high iodide supplementation in maternal rats and their offspring [48]. Other findings show that treatment *in vivo* with an excess of iodide can induce the blockade of thyroid hormone biosynthesis [49] and excess of iodide could induce mononuclear infiltration in salivary gland as recently indicated in a Swiss albino mouse model [50] These results question whether iodide supplementation would work to boost anti-influenza defenses.

In summary, the findings presented here shows that the H_2_O_2_/LPO/(SCN^-^/I^-^) system robustly inactivates a wide range of influenza viruses that are the major circulating serotypes and species causing epidemics and are also components of the annual influenza vaccine. These results are very encouraging considering the likely contribution of this mechanism to *in vivo* influenza clearance and its potential as a target for pharmaceutical intervention.

## Supporting information

S1 Data Plaque forming unit results. The original data of the plaque forming unit assays using MDCK cells of the virus strains tested in this work are shown for each experiment organized according to the figures.
(XLSX)

## Abbreviations

Duox1: Dual oxidase 1
GO: glucose oxidase
HA: hemagglutinin
OI^-^: hypoiodite
OSCN^-^: hypothiocyanite
IAV: influenza A virus
IBV: influenza B virus
I^-^: iodide
LPO: lactoperoxidase
MDCK: Madin-Darby canine kidney cells
NA: neuraminidase
SCN^-^: thiocyanate
